# “I feel free”: Experiences of a dance intervention for adolescent girls with internalizing problems

**DOI:** 10.3402/qhw.v11.31946

**Published:** 2016-07-12

**Authors:** Anna Duberg, Margareta Möller, Helena Sunvisson

**Affiliations:** 1 Faculty of Medicine and Health, Örebro University, Örebro, Sweden; 2 University Healthcare Research Center, Region Örebro County, Örebro, Sweden

**Keywords:** Adolescent health, dancing, non-judgmental, qualitative research, self-trust, stress, togetherness

## Abstract

Adolescent girls today suffer from internalizing problems such as somatic symptoms and mental health problems at higher rates compared to those of previous decades, and effective interventions are warranted. The aim of this study was to explore the experiences of participating in an 8-month dance intervention. This qualitative study was embedded in a randomized controlled trial of a dance intervention for adolescent girls with internalizing problems. A total of 112 girls aged 13–18 were included in the study. The dance intervention group comprised 59 girls, 24 of whom were strategically chosen to be interviewed. Data were analyzed using qualitative content analysis with an inductive approach. The experiences of the dance intervention resulted in five generic categories: (1) An Oasis from Stress, which represents the fundamental basis of the intervention; (2) Supportive Togetherness, the setting; (3) Enjoyment and Empowerment, the immediate effect; (4) Finding Acceptance and Trust in Own Ability, the outcome; and (5) Dance as Emotional Expression, the use of the intervention. One main category emerged, Finding Embodied Self-Trust That Opens New Doors, which emphasizes the increased trust in the self and the ability to approach life with a sense of freedom and openness. The central understanding of the adolescent girls’ experiences was that the dance intervention enriched and gave access to personal resources. With the non-judgmental atmosphere and supportive togetherness as a safe platform, the enjoyment and empowerment in dancing gave rise to acceptance, trust in ability, and emotional expression. Taken together, this increased self-trust and they discovered a new ability to “claim space.” Findings from this study may provide practical information on designing future interventions for adolescent girls with internalizing problems.

Research shows that mental health problems are currently among the biggest public health challenges globally (Ferrari et al., 2013) and affect 10–20% of children and adolescents worldwide (Kieling et al., 2011). The frequency of mental health problems among adolescents is higher than it has been in decades (Bor, Dean, Najman, & Hayatbakhsh, 2014), and girls show a higher prevalence of health complaints compared to boys (Bor et al., 2014; King et al., 2011). The latest cross-national study from the World Health Organization (WHO), Health Behavior in School-aged Children (HBSC) 2013/2014 (World Health Organization, 2016), that surveyed 42 countries in Europe and North America, showed that by age 15, one in five girls reports fair or poor health and 50% experience multiple health complaints more than once a week. Moreover, Sweden was noted as having the largest increase in mental health problems among all HBSC countries. Internalizing problems such as somatic symptoms (headache, stomach ache, and tiredness) and mental health problems (nervousness, stress, and anxiety) among adolescent girls are a growing public health concern (Folkhälsomyndigheten, 2016). It has been shown that girls are more exposed to interpersonal stress, tend to be more sensitive to others’ reactions over their successes and failures (Murberg & Bru, 2004; Rudolph, 2002), and strive to live up to the needs and expectations of others more compared to boys (Wiklund, Bengs, Malmgren-Olsson, & Ohman, 2010).

The gendered patterns of adolescent mental health problems are therefore relevant to consider. Gender, viewed as a social and historical construction created through social interaction, is defined by Connell (2009) as a dynamic process of social relations, norms, and power structures. Moreover, adolescence is an important time also with regard to gender differences in body image concerns (Jones & Griffiths, 2015). A focus on the body's external appearance has negative consequences, as objectification has proven to be a possible predictor of depression among young women (Jones & Griffiths, 2015).

According to the philosophy of Merleau-Ponty (Merleau-Ponty, 2002), we are embodied subjects involved in existence. He describes that we experience the world through our “lived body,” which is experienced both as perceived and perceiving in a dialectical way of living. Our lived body is constantly present in all our sensations, thoughts, communication, and actions as it incarnates the conscious self. In the view of Merleau-Ponty, we perceive the world through our bodies. The lived body is also emphasized in the existential dimension of dance practice (Lindqvist, 2010). Dance has been suggested to strengthen the bodily connection (Grönlund & Renck, 2006), increase psychological well-being (Quin, Redding, & Frazer, 2007), and improve poor body image and physical self-perception (Burgess, Grogan, & Burwitz, 2006) for adolescents. Additionally, for adolescent girls, dance interventions have been shown to increase self-rated health (Duberg, Hagberg, Sunvisson, & Moller, 2013).

It has been recommended that interventions with physical activity targeting health benefits for adolescents should focus on enhancing enjoyment, autonomy, confidence, and social affiliation (Springer, 2013). Furthermore, as intrinsic motivation, for example engaging in an activity for pleasure, is an important aspect to retaining physical activity (Ryan & Deci, 2000); it is important to offer a form of physical activity that is in line with the interests of the intended target group. Dance is a social and cultural form of physical activity that has been reported to be popular with girls and young women (O'Neill, Pate, & Liese, 2011). It is also linked to increased awareness of emotional processing and a higher ability to interpret the emotions of others (Bojner Horwitz, Lennartsson, Theorell, & Ullen, 2015). Dancing seems to facilitate the identification of both adverse and empowering emotions, and thereby increases access to embodied knowledge. The term “embodiment” refers to the body's emotional feedback system through which the motor system is linked to the cognitive affective system (Bojner Horwitz, Lennartsson, et al., 2015; Koch & Fischman, 2011). Embodiment is also described as a construct and a process in which humans simultaneously are social beings and biological organisms (Krieger, 2005). Dance is suggested to capture and embed all aspects of embodiment, as it is an intellectual, emotional, and physical mediator for understanding our bodily way of being-in-the-world (Block & Kissell, 2001).

Experiences during a physical activity like dance can be complex and can include subtle changes in bodily connection, perceptions of the self within a group, gradations of the dance experience, and emotional aspects. Therefore, a qualitative approach is well suited to investigating experiences of this kind of intervention (Verhoef, Casebeer, & Hilsden, 2002).

To our knowledge, no previous studies have investigated adolescent girls’ experiences of participation in an after-school intervention with dance, or specific experiences of dance in a group setting, when experiencing internalizing problems. There is a huge need for studies that increase the knowledge of adolescent girls’ experiences, to let their own voices to be heard. Therefore, the aim of this study was to explore the experiences of participating in an 8-month dance intervention for adolescent girls with internalizing problems.

## Method

### Participants

This study was embedded within a randomized controlled trial (RCT) of dance intervention for adolescent girls with internalizing problems (Duberg et al., 2013), conducted in a medium-sized Swedish city. Inclusion criteria were somatic symptoms such as headache, stomach ache, tiredness, and aching shoulders; and mental health problems such as nervousness, stress, and anxiety. Recruitment was performed in collaboration with school health services; the school nurses communicated with eligible girls and also invited them to an informational meeting. A total of 112 girls aged 13–18 (mean age 16) was included in the study. The dance intervention group comprised 59 girls, 24 of whom were strategically asked to participate in interview.

All participants received written and oral information about the study before signing a written consent. For girls aged <15, written consent was also provided by their parents/guardians. They were furthermore informed that participation was voluntary and confidentiality was guaranteed. The interview files were coded. Participants found to be at risk of severe depression were offered to meet an experienced, licensed psychologist. The Regional Ethical Board in Uppsala, Sweden, approved the study (DNR 2008/134).

### Intervention

The after-school intervention with dance took place at a centrally located gym twice weekly for 8 months, under the guidance of three trained dance instructors (one at a time). It has been described in a previous publication (Duberg et al., 2013). Each dance class lasted 75 min and included: a 15-min warmup, 40 min of dance practice, and 15 min of relaxation including a brief light massage in pairs, rounded off with 5 min for reflection. A main focus of the intervention was upon the enjoyment of movement. The girls’ internalizing problems were not brought up. The dance was mostly choreographed, but improvization and spontaneous movements were always included to encourage creativity. The self-determination theory, with the key components of competence, relatedness, and autonomy (Ryan & Deci, 2000), was the base of the intervention design. The participants also had the opportunity to provide some input into the dance classes regarding music and dance styles. There were no performances; the intention was solely to offer an opportunity to have a positive dance experience without external pressure, to enjoy music, culture and socialization with peers (about 20 girls per group), and to enhance body awareness. At the end of the intervention, the participants were presented with a number of alternatives to keep up their dancing or exercise elsewhere.

### Procedures

Each girl was given a diary at the start of the intervention and was informed that she could write her thoughts down in it, but was not obliged to do so. The diary would neither be collected nor read by anyone but herself, but could be an individual support during the intervention and a basis for the interviews. About 2 weeks before the interviews, the first author (AD) discreetly asked a strategic sample of the girls if they wished to participate in an interview. To ensure heterogeneity, the sample was determined by a variety in aspects such as age, school, and family background as described in Table I. As the aim was to explore different experiences of an intervention, a range in participation was considered important and was ranging from 45 to 94%. Everyone who was asked to participate agreed to do so. They received information about the study and were given the opportunity to ask questions and time to consider the request.

**Table I:** Strategic sample of girls chosen for interview.

Age	Ranged from 14 to 19 (mean (16,5) years old)
School	From nine different schools in the region
Participation	Ranged from 45 to 94%
Dance habits before start of intervention	Ranged from never participated in any dance before to being used to regular dance
Family background	A mixture of different family backgrounds; divorced parents/living with both parents/living with only the mother/parents with psychiatric diagnosis or disorders
Born in Sweden	Born in Sweden: 20 (83%), born outside Sweden: 4 (17%)

The interviews took place in a secluded, homey room at the university research center about 1 week after the dance intervention had ended. The interviews were face-to-face, semi-structured interviews, and all were conducted by the same person, the first author (AD), for internal consistency. At the onset of each interview, the participants were assured of confidentiality and reminded that they were free to terminate the interview at any time. The length of the interviews varied between 35 and 90 min depending on the individual responses of the participants. The interviews were digitally recorded with the participants’ permission. The interviews started with information about the purpose of the study and a short, informal chat to make the girls feel comfortable in the interview situation. The phrase, “Tell me about your experience of participating in the dance intervention,” followed. The interviews were based on open-ended questions from an interview guide (Table II) and participants were encouraged to speak freely about their experiences of the dance intervention. Follow-up questions (e.g., “Could you tell me more?”) were sometimes posed, which is recommended to obtain richer material (Kvale & Brinkmann, 2009).

**Table II:** Interview guide: primary questions.

Why did you participate in the dance project? Tell me about your thoughts about participating, before you started. Tell me about your experience of participating in the dance intervention. What did you like most/least about the dance practice? How does your body feel when you dance? Has it changed? How? How does it feel when you are on your way to the dance class? How does it feel when you are on your way from the dance class? How does it feel to be in a group? Is there anything you have learned about yourself during the dance intervention this year? In what way has participation in the dance project affected your daily life? Is there something more that you want to tell me?

### Data analysis

Interviews were conducted and analyzed with qualitative inductive content analysis, as described in earlier work (Elo & Kyngas, 2008). The verbatim transcription of the interviews was made by an experienced secretary. Each interview was read through by all the authors in the preparation phase. The authors wrote down notes about the content in the margin of the text while reading it repeatedly, in an open-coding process. Data were then analyzed using the NVivo 10 software program (QSR International, 2014). The first author (AD) generated codes and identified categories. The use of NVivo proved to be a valuable tool in the analysis as it made it easier to move between the various parts of the data set. All of the authors (AD, MM, and HS) then met to discuss the coding and generate categories. Based on this process, a coding process was agreed upon and AD coded the remaining interviews. Meaning units relevant to the aim of the study were extracted and the core of the content was condensed to codes. These codes were, through interpretation, sorted and grouped into 20 subcategories, which were intended to reflect the core message of the interviews. To ensure that the content was correctly understood, the process included going back and forth between the different steps, and AD and HS discussed the coding item by item to ensure consistency. Thereafter, the subcategories were sorted into five generic categories. These were aimed to be descriptive and give a representative picture of the text units. Examples of the analysis process are presented in Table III. Finally, the main category was identified through abstraction (Table IV). All of the authors checked for the accuracy of the translations and interpretation of the quotes presented.

**Table III:** Example of the process of the analysis; moving from the text (meaning unit) to codes, subcategories, and generic categories.

Meaning units	Codes	Subcategories	Generic categories
“Because, you know, everywhere in our society it's all about grades or credits, and stuff, and it's so nice to go to dance. Because there you can let go of everything else and, like, just be. Without always trying to achieve something. That's amazing.”—S	Dance offers an oasis from grades and external pressure	Dance as an oasis from grades and external pressure	Dance as an oasis from stress
“That, I mean, there's been such high demands at school sometimes … And then, when you dance like this in your free time, there are no demands at all—it's really nice.” —K1			
“And that it's much better, I mean, it's a way worse feeling when you know that you're always being judged. You know that someone is standing and watching you and is going to, like, rate you.” —S1	It is a horrible feeling to be judged, and to be watched and rated	It is important to have a non-judgmental zone	
“Well, it's great that you've done this, but you could be even better and now you have to do this even better.” Yeah, basically everything is about achieving. […] That's why it's so nice to enter a dance studio where achievement just isn't that important.”—T1	You can always do better and achieve more. Dance serves as a contrast to the achievement-oriented environment		
“When you do a dance you can experience those feelings for yourself.”—A3	Dance embodies emotions	Use dance to identify and communicate emotions	Dance as emotional expression
“There's a feeling you have, and when you then take some (dance) steps you know—you think, ‘Well, this is something I can really relate to!’—You heighten the feeling and really show it.” —M1	Embraces dance moves that she can emotionally relate to		

**Table IV:** The girls’ experiences of the intervention presented in generic categories and main categories.

Generic categories	Main category
1. An Oasis from Stress	Finding Embodied
2. Supportive Togetherness	Self-Trust That
3. Enjoyment and Empowerment	Opens New Doors
4. Finding Acceptance and Trust in Own Ability	
5. Dance as Emotional Expression	

## Results

The central understanding of the adolescent girls’ experiences was that the dance intervention gave access to and enriched personal resources. With the non-judgmental atmosphere and supportive togetherness as a safe platform, the enjoyment and empowerment in dancing gave rise to acceptance, trust in ability, and emotional expression. Taken together, this led to an increased self-trust. This understanding of the girls’ experiences of the dance intervention is presented as five generic categories and one main category in Table IV.

The main category, Finding Embodied Self-Trust That Opens New Doors, is a comprehensive understanding of the girls’ narratives and represents the girls’ experience of an increased “incarnated” trust in themselves and the ability to approach life with a sense of freedom and openness.

In the following, the generic categories are described separately, supported by quotations illustrating how the analysis is grounded in the interview material.

### An Oasis from Stress

On the whole, the girls defined stress as something they lived with every day in the form of perceived pressure from sociocultural norms and critical self-evaluations. They described that it was sometimes challenging to handle internal and external pressure, and they often felt susceptible to the influence of the media. The dance intervention was described as a desirable time out, an activity that the girls longed for during the school day. The undemanding and supportive atmosphere of the intervention served as a contrast to the high pressure and demands the girls experienced elsewhere. The girls said that “to be able to come as you are” was crucial for the motivation to participate.

Because, you know, everywhere in our society it's all about grades or credits and stuff, and it's so nice to go to dance. Because there you can let go of everything else and, like, just be. Without always trying to achieve something. That's amazing.—S

Almost all the girls’ narratives underlined the value of having this oasis and described the importance of a permissive environment for teenage girls today. This zone, free from pressure and expectations, made it possible for the girls to “be themselves” for a while, and feel included without having to deliver and perform. The dance intervention gave them a non-judgmental breathing space and a feeling of being allowed to “lay off the mask” for a while.

I mean, you're supposed to be a good friend; you're supposed to be good at school, be a girlfriend, and be special. But when you went to dance, you didn't have to be someone special—that's just how it was. You didn't have to achieve anything in particular and then, well, that's why I felt safe there—I could just be myself. There, I could relax and be this person—I didn't have to present myself as happy and strong. I could relax and just dance and stuff.—J

As described above, the pressure of keeping up a perfect and “special” appearance went beyond school demands; it also included friends and close relations. It was part of each girl's daily life to feel critically evaluated by peers and/or herself. Participation in the dance intervention provided a protected zone where she could relax, just dance, and feel good enough.

Another aspect of this oasis from stress was the feeling of “being allowed to” move freely. Several girls described that their daily life was, and had been before the intervention, mostly sedentary, and moreover, the physical education offered at school was described as “controlled” and not always in line with their interests. Therefore, the movement in the creative dance provided a newfound feeling of freedom.

Free. Because in real life it's not often that you can really let go—throw out your arms, run around, leap and twirl around like that. I feel free.—A2

In this quotation, this interviewee made a distinction between the experienced reality, which was mostly sedentary or controlled, and the intervention, which were experienced as a secure oasis where creative movements were allowed and gave room for joyful bodily expressions. This was described as a concrete source of stress reduction.

### Supportive Togetherness

The importance of supportive social togetherness was mentioned in all narratives. Also, this aspect was considered crucial for participation. Connecting with others, who had similar internalizing problems and experienced the same pressures and demands, contributed to feeling comfortable in the dance group setting. The dance intervention gave the participants the comforting insight that they were not alone with these problems, and it also provided the opportunity to meet new friends from other schools. Some of the girls had a history of being lonely.

And it's, like, after ten years you're pretty tired of always being by yourself, doing nothing and, like, hanging around at home. And suddenly you're seeing friends who actually seem to want to spend time with you and then it's a whole different thing—you start to develop as a person. So you find yourself in a whole new situation.—S2

This new experience of social community gave a feeling of being included and being welcome, which, as the above girl mentioned, led to personal development. Another girl described how harshly judgmental the tone between girls can be in different group settings, and how easy it is to criticize oneself for failing to meet perceived expectations in these competitive environments. As a contrast, this participant experienced the girls-only environment of the dance intervention as entirely supportive.

And there was a lot like jealousy and snide remarks, and people watching each other to see what the other person was doing. It wasn't really like people were supporting each other, it was more a competitive thing among girls … But in the dance project, it was really the opposite of that, like, I have never met such a big group of girls in which everyone feels, like “No, we support each other. This is not about competition. We can just relax and be ourselves here.” So that was a bit of a shock—it really was.—J

For this girl, the togetherness in the dance group that emphasized acceptance and kindness was unique. The interviewees acknowledged that, initially, at the beginning of dance intervention, there undoubtedly was social insecurity, but togetherness and the permissive atmosphere contributed to a rapidly developing feeling of acceptance and friendly inclusion. Competitive comparison diminished as the connection to others in the group improved and togetherness became more prominent. Several girls noticed that the less time they spent comparing themselves to others in the dance group, the more time they spent actually making new friends. The newfound acceptance and the ability to share the experience with similar others were described as a source of joy.

If you make a wrong move, you just look at each other and laugh your head off. So I thought it was really fun to be in a group, and not have to dance alone.—A1

The narratives revealed that the supportive togetherness experienced in the dance intervention contributed to an overall reduced focus on the culture of constant evaluation they experience and are part of, even in other social groups. Almost every girl highlighted the value of a sense of belonging and making new friends, and described how the newfound friendships lasted even after the intervention had ended.

### Enjoyment and Empowerment

Another aspect that was highlighted by almost every girl was that dancing gave rise to a feeling of enjoyment.

It feels like, you know, you're breaking free from what you've done earlier that day and really go in there and give it all you've got, here I don't have to think about anything else, just be happy and give it all I got, and you know, have fun with everybody else. —A2

Over the intervention period, negative thoughts of self-doubt or incompetence were increasingly replaced with feelings of accomplishment, having fun, and being in control. The creative part of the dance session was described as playful and was useful for exploring new movements. The rhythm, grace, strength, balances, and posture in the dance were described as concrete new skills. The girls stated that they had successively learned to enjoy different kinds of dance moves and thus put more trust in their bodies.

Plus, as soon as I get dancing I feel like, “This is me. I'm standing on my own two feet and I can do whatever I want. No-one else can come and tell me what I should be doing.”—A1

The above quotation is a straightforward description of this girl's increased empowerment and body awareness, as well as her sense of integrity. Several girls gave examples of how dancing helped them to explore their bodies as positive resources. Given the extensive time period of the intervention, this newfound connection to their bodies had enough time to consolidate. Some dance moves were described as being “incarnated” as they learned to believe in their ability and to claim more space in the room:

Yeah, but if you feel good about yourself you automatically take up more space—maybe not physically, but you have the attitude that “I'm here—I can actually do something. I'm good at something, or I perform … I can dance. Even if I'm not that great, I can dance” … and then it's like, well, you stand a bit taller. Yep, I think you really do take up a little more space.—C

The girls described the newfound, powerful feeling of dancing as an energizing and vivid experience. Many of the girls described the experience of empowerment that arose during and after the dance session as energizing, and saw the music as an extra dimension that underlined the arousal and enjoyment of the dance practice. They often stated that this feeling was hard to explain, but examples can be found in the following quotations:

Because—and this is also like after a workout—I love it when I'm all sweaty and flushed, like, I just get so much energy … I get so stubborn and angry … I turn into the girl I want to be.—J

It's, like, you've sweated out all the old shit you bring from school or whatever, and a whole new person opens up inside. I don't really know what it is, but that's how it feels to me in any case.—A1

Several girls described “aha moments” when they suddenly realized that they had shifted focus in how they perceived the dance move; from objective self-consciousness to a subjective focus on the dance and music.

At first I looked at myself—God! I'm moving my arm wrong; it looks really weird when I do it, all different from how everyone else is doing it. But now it's like; “But this is such a nice movement!”—A1

This quotation illustrates a newly found confidence that was anchored in the body and that allowed this participant to focus entirely on her own experience rather than on what others might think. This confidence gave rise to a sense of full “absorption” in the dance, which was described as a foundation for experiencing enjoyment and empowerment.

It is notable that for many of the girls, getting to the dance class after a long day at school was sometimes challenging and tiring. However, this did not stop them from coming and they described that, as soon as they stepped into the changing room, they had entered new positive emotional state, which grew stronger once the music started playing.

### Finding Acceptance and Trust in Own Ability

All the narratives highlight examples of how the girls were more likely to experience and express positive feelings and thoughts about themselves. A more non-judgmental, compassionate attitude emerged. Not having to be perfect all the time gave space for increase of personal acceptance:

And I think that, more than anything, I've stopped looking at everything as being so emotionally charged—like, things don't have to be perfect. There's no need to be perfect all the time. You can do things that make you feel good instead.—M

Although they sometimes experienced a struggle, overall, the girls described a more human approach as they embraced a more accepting attitude towards imperfections, in both themselves and others. This was contingent with being able to see nuances in perceived failures in everyday life, and more often accept setbacks in life as natural events:

If I have setbacks, I know that, like, hey, it's going to be ok … things may be tough sometimes, it's always like that, but they do get better.—A2

The girls expressed relief about the accepting attitude, which allowed lower expectations and demands for performance. Statements such as, “It's going to be ok,” were commonly expressed, together with a positive view of the future.

Moreover, they described a newfound trust in their own abilities. They saw themselves as capable of taking new steps in life, such as starting new leisure-time activities, traveling, studying, taking part in social events, making their voice heard in a group, or, in the dance intervention, attempting a new, more challenging choreography or creating spontaneous dance moves without self-doubt.

I became proud of myself, you know? Like, “Wow! Here's another thing I can do! I can! I don't have to be so prepared for everything. I can trust myself … trust my own ability.”—C2

Accordingly, this newfound trust in own ability that the girls described was not contingent on how they measured up to others. Rather, it was based on a growing feeling of personal competence. This seemed to contribute to being able to face new situations in life with confidence.

You find yourself able to ask for what you need—anywhere, in any situation. And I think you get better at handling new situations, because dance changes all the time—yeah, it makes you better prepared to deal with all the different things that happen in your life.—S4

### Dance as Emotional Expression

A central aspect of the dance intervention experience was how different dance choreographies enabled affirmation of different kinds of emotions. The ability to express emotions through dance movements, instead of words, facilitated acceptance and justification of these emotions. An enriched body awareness and body language facilitated expression of feelings, such as being angry, sad, happy, free, and powerful, and being in harmony with others.

It's like, you don't have to go up to that person and say, “I'm really angry with you,” to get it out. Instead, you can dance—you can get it out that way.—M

The ability to use dance movements as a new form of expression was experienced as a possibility to identify, recognize, and communicate different kinds of everyday emotions in dance. This was described as both comforting and strengthening, and one girl described it as “a new language” that was closer to her:

So I feel that … I always feel so much better, I feel so up …, once I get dancing …. I feel that dance is like another language—it's like, the person you really are is expressed in the movements. So it's like a whole new thing, I think. I really love expressing myself in this way.—A1

As illustrated by the above quotation, this girl's bodily expressions constituted a source of joy and satisfaction. According to the girls, acknowledging both positive and adverse everyday emotions in dance was a way of giving voice to different mental states. They gave several concrete examples. One was a dance choreography that included falling to the floor several times, and the falls were spontaneously called “dying.” This somewhat dramatic, but playful setting was appreciated, and one of the girls translated this right into her life.

It was in this Marilyn Manson dance …. I said, “When we die, it's like life … you fall and you get up again.” And right away, it made me think about how life has been for me: I've fallen, but I've got up again.—M

This movement helped this girl to accept the fall and to focus on the positive, elevating part of the movement. Thus, this point to how dance can enable identification, validation, and interpretation of emotions.

## Discussion

The analysis resulted in five generic categories and one main category, titled Finding Embodied Self-Trust That Opens New Doors. The main category represents the girls’ experience of an increased trust in the self and the ability to approach life with an enriched sense of freedom and openness. In all, this can be understood as a way of perceiving the resourcefulness of the self. This recast and enriched perception of the self and life that the girls described can be explained as having emerged from the fact that, when using the body in new ways, a person can learn to see things differently (Anderzen-Carlsson, Persson Lundholm, Kohn, & Westerdahl, 2014).

One of the essential meanings of the main category is for each individual participant to develop the ability to trust in her right to “claim space.” The new experiences and personal development increased the participants’ capacity to access life with a sense of freedom and an open approach. This openness has also been reported in a basic body awareness therapy intervention by Danielsson and Rosberg (2015), who identified the theme “opening toward life” in their analyses. To “claim space” in this sense is also described by the participating girls in Strombacks’ (2013) stress management course, in which the young women described the processes of bodily and personal empowerment as embracing the possibility of “claiming space,” and taking a step forward or withdrawing from negative or demanding attention, both physically and metaphorically. The feeling of being free and open is repeatedly highlighted in the current narratives, an interesting contrast to the theorizing about girls’ and women's inhibited movements and the restriction of embodied vitality in their daily lives. The girls’ narrated experiences challenged the gendered patterns of “living up to the expectations of others” as they stressed the importance of being able to enjoy dance and the dance movements solely for its own sake, not for evaluation by others or to rehearse for a show. This illuminates the focus shift from a viewpoint of objectively observing movements of the body to subjectively experiencing them from within. Given that many girls and women, today, feel compelled to constantly monitor their appearance to live up to society's standards; embodied experiences can potentially provide balance by creating an inside perspective.

In the current narratives, the girls described a newly acquired confidence that was anchored in their bodies. They explored new ways of using their bodies to express themselves and learned to embrace their full movement potential. Therefore, their movement patterns, their postured and how they moved, can be understood as empowered. It might be considered noteworthy that the girls’ internalizing problems did not seem to be an obstacle to this journey of new embodied empowerment. Although the girls initially often had tense, and sometimes problematic, movement patterns (resulting from internalizing problems), the main category, Finding Embodied Self-Trust That Opens New Doors, can also be understood as introducing the girls to a greater variety of movement patterns and posture. One possible explanation to this positive body-anchored experience in dance may originate from the theory of the emotional brain by LeDoux (1996), who highlights the notion that dance can “surprise” the cognitive brain unconsciously. Emotionally loaded visual and auditory stimuli evoke activities in the emotional brain much more rapidly than in the cognitive brain; thus, dance activities can surpass automated thinking and create new “pathways” that can trigger the participants’ awareness of different emotions and increase well-being (Bojner Horwitz, Grape Viding, et al., 2015).

Another essential aspect of the narratives was the ability to feel “absorbed” by the dance movements. This gave a concrete feeling of being in the present moment. Gyllensten (2010) highlight that bodily experiences are always experienced in the present moment, which is seen as essential for getting in contact with emotions. This is consistent with the philosophy of Merleau-Ponty (2002), who states that the lived body is present in every movement and that it is the unity of body and mind that completes every moment of existence. Furthermore, Merleau-Ponty demonstrates that it is through the body that we are related to, and constantly engaged with, the world and life itself. Thus, by embodying experiences through dance, as highlighted in the main category, Finding Embodied Self-Trust That Opens New Doors, the girls may experience an enrichment of their innate resources in the light of embodied trust in the self in the present moment.

A salient finding in this study is that the dance sessions played a part in how the girls could use their embodied self-trust and enriched body awareness as a stepping stone to a newly won positive attitude towards themselves and others. This is consistent with Danielsson, Hansson Scherman, and Rosberg (2013), who described increased body awareness as an opportunity to encourage an embodied self-trust, and find ways to withstand and manage anxiety symptoms. Moreover, Gyllensten, Skar, Miller, and Gard (2010) suggest that bodily experiences and reflections can lead to a more positive experience of the body and self, and that the basis for self-confidence and well-being lies in the ability to understand one's own emotions and needs through the awareness of the body. The authors describe body awareness as inseparable from the identity and, consequently, as an important aspect for the embodied self in interaction with others. This is confirmed by research showing that body awareness-enhancing therapies may provide psychological benefits for patients suffering from a variety of conditions (Mehling et al., 2011).

It is also important to bear in mind that getting to the dance classes after a long day at school was sometimes described as tiring for the girls in this study. They described this as inconsequential because it did not stop them from attending and they found it enjoyable as soon as they got to the changing room. Nevertheless, it is worth considering that some adolescent girls within this target group of internalizing problems are high-performing girls at risk of disregarding bodily signals.

The girls described the dance experiences as meaningful and their stories expressed an embodied self-trust, which can be understood as meaning that they now trusted their ability and their right to open new doors in life. Examples go beyond the previously discussed ability of taking more space and taking on and creating new choreographies with an empowered movement pattern when dancing. In addition, the embodied self-trust also involved different forms of personal development in the girls’ narratives. The girls began to participate in social events more often, dared to make their voices heard more often, and revealed plans to travel and study in other cities.

Building on the study's five generic categories, we would like to suggest some possible explanations for the newly won embodied self-trust the girls experienced:

The category, An Oasis from Stress, was shown to represent a fundamental basis of the intervention and was important for adherence motivation. To be able to “come as you are” without having to “perform,” was shown to be of particular value for the girls. The fact that they described the intervention as an oasis from the stressful internal and external pressure that they dealt with on a daily basis is noteworthy. It is possible that this permissive non-judgmental atmosphere represented a time out from perceived demands and gendered sociocultural norms, which might have facilitated emphasizing strengths and developing resources.

Another key aspect that proved to be of particular importance for the girls was the supportive social context described in the category, Supportive Togetherness. This is in line with other studies of adolescent girls that show that supportive relationships can have a stress-preventive impact (Schraml, Perski, Grossi, & Simonsson-Sarnecki, 2011), and a sense of unity with others can add meaning and strength to adolescent girls’ life (Larsson, Sundler, & Ekebergh, 2013). This is also concurrent with Stromback (2013) who proves that, in their stress management course, sharing new experiences with “similar others” improved social interaction skills. The dance activity and training itself may also have contributed to the togetherness as dance has been shown to be involved in the body's emotional interplay with others (Bojner Horwitz, Lennartsson, et al., 2015). Overall, however, regardless of age or gender; being engaged in one or more organized, group leisure-time activity is associated with higher life satisfaction and better self-rated health (Badura, Geckova, Sigmundova, Van Dijk, & Reijneveld, 2015). According to the narratives, the combination of social support and reduced focus on competition described contributed to a feeling of being included. This is consistent with Knowles, Niven, and Fawkner (2011), who report that, in their adolescent study population, a positive environment reduced self-presentational concerns, increased enjoyment, and subsequently counteracted the decrease in physical activity behavior. Further regarding positive atmosphere; in our study, the “girls-only” environment was shown to be supportive. This has also been highlighted by other intervention studies (Berry, Kowalski, Ferguson, & McHugh, 2010; Pearson, Braithwaite, & Biddle, 2015; Stromback, Malmgren-Olsson, & Wiklund 2013).

Concurrent with the results described in the category Enjoyment and Empowerment, other studies also highlight enjoyment as an important aspect for girls’ participation in dance (Edwards et al., 2016; Gardner, Komesaroff, & Fensham, 2008). The narratives in this study express a journey from often being tense and insecure, to being more in contact with joyful feelings, which can be valuable considering the internalizing problems. In general, adolescent girls tend to pair “having fun” with “having friends during the activity” and “participating in activities where skill is not emphasized” (Yungblut, Schinke, & McGannon, 2012). Further, it is possible that the empowered movement pattern described in this category helped the girls to feel better prepared to take on new challenges and endure difficult situations. This type of positive experience resulting from dance participation has been shown in other intervention studies (Aktas & Ogce, 2005; Gardner et al., 2008). Empowerment processes have been significantly associated with health outcomes, including self-care behaviors (Spencer, 2014). Furthermore, movement patterns and posture have been suggested by Carney, Cuddy, and Yap (2010) to have the potential of significantly altering mental states and improving well-being. Taking up more space (expansiveness) and keeping the limbs open (openness) is called “power posing” and has been reported to make individuals feel more powerful and “in charge.”

Finding Acceptance is a sign of increased self-compassion (defined by Neff, 2003, as self-kindness and acceptance of perceived imperfections, limitations, and failures). This acceptance contributed to less social comparison. Spending less time making comparisons with others in the group gave the girls a more non-judgmental attitude and made it easier for them to be gentle with themselves. This translated to other settings outside the intervention, which is consistent with findings reported by Rogers and Ebbeck (2016). Since adolescence is a sensitive developmental period when the foundation for later mental health is formed (Kinnunen, Laukkanen, & Kylma, 2010), an accepting attitude can be a valuable recourse when facing setbacks (Neff, 2003).

Finding Trust in Own Ability seems to have played an important role in the girls’ increased self-trust. Trust in one's own ability, self-efficacy, is considered to be central to adolescent girls’ participation in physical activity (Springer, 2013), and, in this case, it seemed to increase trust in personal competence and mastery, not only when dancing but also in other situations.

Finally, regarding the category Dance as Emotional Expression, the ability to use dance movements as a new form of expression was a way of giving more space to the authentic self. Giving voice to different mental states increases the physical connection with emotions and helps with dealing with the challenges in daily life by identifying, recognizing, and affirming different kinds of emotions. This is in line with results from a cultural intervention for women with exhaustion symptoms, which showed an increased awareness of feelings and sensations after the intervention and, furthermore, improved the women's ability to describe and identify feelings (Bojner Horwitz, Grape Viding, et al., 2015).

Taken together, a relationship between the categories emerged in the analysis, as the narrated experiences could be seen in a pattern. The first category An Oasis from Stress was described as a fundamental basis for participation, together with Supportive Togetherness, that represents the important group setting. As this was ensured, the girls could engage in the immediate effect of the dance, Enjoyment and Empowerment, and experience the outcome, Finding Acceptance and Trust in Own Ability, and, moreover, the ability to use Dance as Emotional Expression. To summarize, these experiences gave rise to the main category, Finding Embodied Self-Trust That Opens New Doors; as it seemed to both contribute to individual development and also contribute to increase the girls’ ability to take part in new events in life. The detection of embodied strengths might have reduced the girls’ focus on their internalizing problems and increased trust in their ability to cope with stressful situations. These findings suggests that, in spite of several potential challenges in engaging in a creative physical activity when having internalizing problems, this sample of adolescent girls seemed to benefit from this dance intervention.

### Limitations

A period of 8 months undoubtedly has a great impact on an adolescent girl's life. Possibly, the key reason that the girls credited much of their personal development to the effects of the dance intervention is that new events in adolescents’ lives focus a lot of their attention. An intervention that takes place twice weekly for 8 months obviously demands focus, especially if it is conducted in a new group setting. As 8 months represent a whole school year in this evolutionary time of adolescence, different kinds of neurological, psychosocial, and social factors influence adolescents’ personal development. It is important to consider that the individual growth reflected in the results is a product of many different components. However, as extensive interventions aimed to strengthen health effects are warranted and physical activity declines during adolescence (Nader, Bradley, Houts, McRitchie, & O'Brien, 2008), this type of intervention could be one way of supporting positive lifestyle habits.

### Methodological considerations

A qualitative approach is considered appropriate for exploring and attributing meaning to the participants’ subjective experiences during interventions (Cramer et al., 2013). Collaboration among researchers and peer debriefing was aimed to give verification and trustworthiness to the analysis process. To strengthen dependability, the same interview guide was used in all interviews. A detailed description of the setting, selection, and characteristics of the participating girls has been included to enhance transferability.

The fact that the first author (AD) was both the interviewer and one of the dance instructors facilitated the building of trust and gave us an in-depth understanding of the data. This enabled the girls to feel secure in the interview situation. Obviously, however, there is a risk of bias as they were aware of the interviewer's involvement in the intervention. It is possible that they felt subordinate or wanted to show gratefulness to the research team. For this reason, the girls were informed repeatedly about their right to decline participation in the interview, both when being asked to participate and when being contacted to make an appointment. To reduce the risk of socially desirable answers, all the interviews were deliberately scheduled post-intervention, when the instructor–participant relation had ended. Moreover, the girls were informed that the interviews were not an evaluation of the intervention, but a story of their experiences of the intervention over the past year. However, the given interview structure was discussed repeatedly with experienced colleagues, and the interview process started off with three pilot interviews followed by evaluation. The interviews proved to be rich, and the data collected felt trustworthy, especially as they are in line with other data from the same RCT study (i.e., the positive overall experience of the intervention and mental health effects).

No early drop-outs from the intervention were included in this interview study. To further broaden the insight into different experiences and give new nuanced knowledge about the phenomena, this would be interesting. However, in this study, the purpose was to investigate the experience of the intervention of 8 months, and, logically, those who dropped out after 1–2 weeks were not included. As stated earlier, to ensure the heterogeneity in the sample, we carefully chose to include a generous range in participation adherence, including girls with a low participation rate of 45%.

In this study, qualitative content analysis was used to analyze the data and peer debriefing was always used to ensure credibility. The analysis was repeatedly discussed among all the authors. For trustworthiness, all authors critically reviewed each step in the analytical process. The preconceptions of the researcher, such as previous personal and professional experiences, and theoretical perspectives related to education and interests are of importance in qualitative research (Malterud, 2001). The first author (AD) is a physiotherapist in child and adolescent psychiatry with focus on mind–body therapies. However, the overall intention was to bracket the researchers’ pre-understanding (Creswell, 2007). Both of the co-authors, one of whom is a physiotherapist and the other who is a nurse, had a supervisory role in the study and both had insight into the intervention.

### Further research

This kind of dance intervention is now being implemented in several cities in Sweden, and there is a need for further qualitative research to explore girls’ experiences of the intervention in other settings. Future research should also focus on the findings from qualitative studies on the experiences of different forms of body awareness interventions, as there seem to be many similar experiences, irrespective of diagnosis and intervention type. Moreover, it would be interesting to explore the perceived experiences of this kind of dance intervention for different age-groups in a healthcare setting, such as primary care or child and adolescent psychiatry.

### Implications

The girls’ experiences of the dance intervention indicate the need to develop undemanding, non-competitive physical activity interventions that provide a supportive environment and focus on the joy of movement. Body-anchored interventions that complement school health care can help facilitate and possibly broaden the range of existing interventions in an after-school setting. A non-judgmental atmosphere and supportive togetherness were shown to be of importance for participation, which may provide information about practical implications. Findings from this study may be of value for school health care staff, teachers, and caregivers (both in primary care and in child and adolescent psychiatry) on designing future interventions for adolescent girls with internalizing problems.

## Conclusion

The central understanding of the adolescent girls’ experiences was that the dance intervention gave access to and enriched personal resources. With the non-judgmental atmosphere and supportive togetherness as a safe platform, the enjoyment and empowerment in dancing gave rise to acceptance, increased trust in ability and a space for emotional expression. Taken together, according to our analysis, this led to increased embodied self-trust and an ability to approach life with a sense of freedom and openness. These findings may provide practical information for healthcare providers regarding which aspects might be of value in designing interventions that target the challenge of reducing the burden of internalizing problems for adolescent girls. As described in this study, dance could constitute one example of a non-pharmacological intervention with promise as a complementary treatment. This type of intervention might also motivate this target group to engage in active and positive forms of self-care, thereby alleviating the healthcare workload and contributing to sustained healthy habits.
